# Italian report on RARE epilepsies (i‐RARE): A consensus on multidisciplinarity

**DOI:** 10.1002/epi4.13020

**Published:** 2024-08-23

**Authors:** Antonella Riva, Antonietta Coppola, Francesca Bisulli, Alberto Verrotti, Irene Bagnasco, Maurizio Elia, Francesca Darra, Simona Lattanzi, Stefano Meletti, Angela La Neve, Giancarlo Di Gennaro, Isabella Brambilla, Katia Santoro, Tommaso Prisco, Francesca Macari, Antonio Gambardella, Carlo di Bonaventura, Simona Balestrini, Carla Marini, Dario Pruna, Giuseppe Capovilla, Nicola Specchio, Giuseppe Gobbi, Pasquale Striano, the iRARE Study Group, Bartolini Emanuele, Bonanni Paolo, Boni Antonella, Briatore Eleonora, Canafoglia Laura, Del Negro Ilaria, Fortunato Francesco, Galli Rosita, Giordano Lucio, Giugno Alessia, Giuliano Loretta, Lavra Loredana, Luchetti Anna, Maretti Michela, Marino Simona Domenica, Mastrangelo Mario, Messana Tullio, Parente Eliana, Pellino Giuditta, Peruzzi Cinzia, Piccioli Marta, Pietrafusa Nicola, Pironi Virginia, Prezioso Giovanni, Prisco Giulia, Pulitano Patrizia, Ragona Francesca, Rizzi Romana, Rosati Eleonora, Ruta Maria Rosita, Sarajlija Jasenka, Spagnoli Carlotta, Spalice Alberto, Terrone Gaetano, Trabacca Antonio, Valerio Massimo, Zucca Claudio

**Affiliations:** ^1^ Department of Neurosciences Rehabilitation, Ophthalmology, Genetics, Maternal and Child Health (DINOGMI) University of Genoa Genoa Italy; ^2^ Epilepsy Center, Department of Neuroscience, Odontostomatology and Reproductive Sciences Federico II University of Naples Naples Italy; ^3^ IRCCS Istituto Delle Scienze Neurologiche di Bologna Full Member of the ERN EpiCARE Bologna Italy; ^4^ Department of Biomedical and Neuromotor Sciences University of Bologna Bologna Italy; ^5^ Department of Pediatrics University of Perugia Perugia Italy; ^6^ Division of Neuropsychiatry Epilepsy Center for Children, Martini Hospital Turin Italy; ^7^ Unit of Neurology and Clinical Neurophysiopathology Oasi Research Institute‐IRCCS Troina Italy; ^8^ Unit of Child Neuropsychiatry, Department of Engineering for Innovation Medicine University of Verona, Full Member of the ERN EpiCARE Verona Verona Italy; ^9^ Neurological Clinic, Department of Experimental and Clinical Medicine Marche Polytechnic University Ancona Italy; ^10^ Neurophysiology Unit and Epilepsy Centre, OCB Hospital, AOU Modena Modena Italy; ^11^ Department of Biomedical, Metabolic and Neural Science University of Modena and Reggio Emilia Modena Italy; ^12^ Department of Basic Medical Sciences, Neurosciences and Sense Organs University of Bari Bari Italy; ^13^ IRCCS NEUROMED Pozzilli Isernia Italy; ^14^ Dravet Italia Onlus Verona Italy; ^15^ Epag ERN EpiCare Verona Italy; ^16^ Research Center For Pediatric Epilepsies (CREP), Department of Surgery, Dentistry, Paediatrics and Gynecology University of Verona Verona Italy; ^17^ Alleanza Epilessie Rare e Complesse Italy; ^18^ Associazione Famiglie LGS Italia Correggio Italy; ^19^ Or.S.A Treviso Italy; ^20^ Associazione Sclerosi Tuberosa Rome Italy; ^21^ Department of Precision Medicine University of Campania “Luigi Vanvitelli” Naples Italy; ^22^ Department of Human Neurosciences, Policlinico Umberto I Sapienza University of Rome Rome Italy; ^23^ Neuroscience Department Meyer Children's Hospital Florence Italy; ^24^ University of Florence Florence Italy; ^25^ Department of Clinical and Experimental Epilepsy UCL Queen Square Institute of Neurology London UK; ^26^ Child Neurology and Psychiatry Unit Children's Hospital “G. Salesi” Azienda Ospedaliero‐Universitaria Delle Marche Ancona Ancona Italy; ^27^ Pediatric Neurology and Epileptology Unit, Pediatric Department ARNAS G. Brotzu/ASL Cagliari Italy; ^28^ Child Neuropsychiatry Department, Epilepsy Center “C. Poma Hospital” Mantova Italy; ^29^ Fondazione Poliambulanza Brescia Italy; ^30^ Neurology, Epilepsy and Movement Disorders Unit, Bambino Gesù Children's Hospital, IRCCS Full Member of European Reference Network EpiCARE Rome Italy; ^31^ IRCCS Istituto “Giannina Gaslini,” Full Member of the ERN EpiCARE Genoa Italy

**Keywords:** DEEs, Delphi, management, multidisciplinarity, rare epilepsies

## Abstract

**Objective:**

Rare and complex epilepsies encompass a diverse range of disorders characterized by seizures. We aimed to establish a consensus on key issues related to these conditions through collaboration among experienced neurologists, neuropediatricians, and patient advocacy representatives.

**Methods:**

Employing a modified Delphi method, a scientific board comprising 20 physicians and 4 patient advocacy representatives synthesized existing literature with their expertise to formulate statements on contentious topics. A final 32‐member expert panel, representing diverse regions of Italy, validated these statements through a two‐round voting process, with consensus defined as an average score ≥7.

**Results:**

Sixteen statements reached a consensus, emphasizing the necessity for epidemiological studies to ascertain the true prevalence of rare epilepsies. Etiology emerged as a crucial factor influencing therapeutic strategies and outcome prediction, with particular concern regarding prolonged and tonic–clonic seizures. The importance of early implementation of specific drugs and non‐pharmacological interventions in the treatment algorithm for developmental and epileptic encephalopathies (DEEs) was underscored. Multidisciplinary care involving experts with diverse skills was deemed essential, emphasizing non‐seizure outcomes in adolescence and adulthood.

**Significance:**

This national consensus underscores the imperative for personalized, comprehensive, and multidisciplinary management of rare epilepsies/DEEs. It advocates for increased research, particularly in epidemiology and therapeutic approaches, to inform clinical decision‐making and healthcare policies, ultimately enhancing patients' outcomes.

**Plain Language Summary:**

The modified Delphi method is broadly used to evaluate debated topics. In this work, we sought the consensus on integrated and social care in epilepsy management. Both representatives of high‐level epilepsy centers and patients' caregivers were directly involved.

**Key points**

- Rare and complex epilepsies are heterogeneous disorders with “seizures” as a common hallmark.
- We used a modified Delphi approach to seek consensus on debated topics in DEEs management on the national territory.
- Multidisciplinary and social care emerged as pivotal to guarantee proper treatment.

## 1 INTRODUCTION

Rare and complex epilepsies, although individually rare, come to affect nearly 5 people in 10 000.^1^ They comprise conditions with etiological and phenotypic heterogeneity brought together by seizures, which often prove to be resistant to polypharmacotherapy thus influencing neurodevelopmental trajectories, quality of life (QoL), and mortality. To address these facets, the International League Against Epilepsy (ILAE) coined the wording Developmental and Epileptic Encephalopathies (DEEs).^2^ The conjunction of the two terms “Developmental” and “Epileptic” emphasizes that brain construction processes (including synaptogenesis and neuronal sprouting) may be early distorted by the underlying disease cause (i.e., genetic) on which seizures superimpose.^3^

Dravet Syndrome (DS), Tuberous Sclerosis Complex (TSC), Angelman Syndrome (AS), or Cyclin‐dependent kinase‐like 5 deficiency disorder (CDD) have well‐defined causative mutations in neuronal expressed genes. The α1‐subunit of the voltage‐gated sodium channel (*SCN1A*) was the first epilepsy‐associated gene, discovered in the early 2000s.^4^ Pathogenic variants in *SCN1A* are found in about 80% of DS patients and they commonly lead to a loss‐of‐function (LOF) of the channel properties.^5^ As one of the most common monogenic epilepsies, the natural course of DS has been dissected over the years. DS is characterized by fever‐induced alternating hemiclonic or convulsive seizures around the 5th month of age.^6^ The increased number of drug‐resistant seizures, with a risk for prolonged convulsive seizures or status epilepticus (SE), invariably influences psychomotor development and the adolescent/young adult with DS typically retains intellectual disability and psychobehavioral problems.^6^ TSC may feature symptoms of a common DEE. Although seizures strongly affect the everyday life of patients and their caregivers, mutations in the tumor‐suppressor genes *TSC1* or *TSC2* make it a multisystemic disorder. Patients may develop hamartomas in different organs^7^; moreover, about 50% of young children have autism spectrum disorder (ASD) or attention‐deficit/hyperactivity disorder (ADHD) getting to talk about TSC‐associated neuropsychiatric disorders (TAND).^8^, ^9^ AS is due to the loss of function of the imprinted *UBE3A* gene, which can result from four different mechanisms that make the maternally inherited gene non‐functional.^10^ The main features of the disease are ataxia, intellectual disability, lack of speech, seizures, hyperactivity, and uncontrolled laughter.^11^ Epilepsy persists in more than half of adults with AS, however visual, mobility, and behavioral disorders are also present and need appropriate care.^12^ CDD shares some phenotypic features with Rett syndrome but has distinguishable symptoms and a clear genetic culprit: the X‐linked *CDKL5* gene, coding for a protein involved in brain development.^13^ Beyond seizures, CDD patients (mostly girls) display different degrees of developmental delay, autistic‐like symptoms, sleep disturbances,^14^ and cerebral visual impairment as a constitutive part of the disorder.^15^

Lennox–Gastaut Syndrome (LGS) is classified by the ILAE as a DEE, although its definition is based on electro‐clinical features rather than on etiology; that is why the LGS group comprises different rare and complex diseases with genetic and/or structural pathogenesis. Several efforts have been made by epileptologists to uniform its definition.^16^, ^17^

Consequently, the traditional concept of “comorbidities” in rare and complex epilepsies is quite outdated. Instead, psychobehavioral and movement disorders are recognized as integral components of these conditions. This paradigm shift has fundamentally transformed the approach to DEEs in recent years.

There is increasing awareness of the need for a multidisciplinary approach, which includes the involvement of various clinical care professionals such as physiotherapists, neuropsychomotor therapists, psychiatrists, and psychologists. This holistic approach, to be brought into adulthood, is crucial for enhancing the QoL, mitigating complications, and optimizing treatment outcomes.^18^, ^19^ Our objective was to establish an Italian Consensus, drawing upon the perspectives of both caregivers and seasoned physicians, to address contentious issues in the realm of rare and complex epilepsies.

## 2 METHODS

The consensus procedure followed a modified Delphi approach designed to address the appropriateness of healthcare interventions. This method generates statements of constructed scenarios by integrating scientific evidence with the experience and judgment of a multidisciplinary group of experts. It serves as a model to produce best‐practice recommendations in situations where scientific evidence or experiences are limited, contradictory, or not well integrated into clinical practice.^20^, ^21^

The Scientific Board (SB) was composed of 20 physicians selected based on their expertise in rare and complex epilepsies and scientific publications. Ninety‐five per cent of them had >10 years of experience and seven were representatives of five recognized Italian EpiCare Centers.^1^ The representation of the whole national territory was also taken into account. The board also included a representative of each of the following family advocacy groups: Dravet Italia Onlus; Associazione Famiglie LGS Italia; Organizzazione Sindrome di Angelman Onlus (OR.S.A); and Associazione Sclerosi Tuberosa. All the meetings of the consensus procedure took place in virtual mode. In the first meeting (May 18th, 2023), the SB discussed different aspects of rare epilepsies by analyzing the published literature. The number of statements was not predefined, and they were considered useful to clarify, in the opinion of the experts, the unmet needs of the above‐mentioned topics. Following extensive discussion among SB members, 16 statements were formulated. From May to October, a larger, anonymized, group of national experts sought consensus on these statements.

In the first round of voting, the clinicians of the extended working group were individually asked to express their judgment of appropriateness, using a 9‐point scale ranging from 1 (not appropriate) to 9 (very appropriate), encompassing 4–6 (appropriateness uncertain). After the first voting round, the anonymized votes were analyzed by study facilitators and mean values ±SD were calculated. Areas where consensus was not achieved were re‐proposed to the SB.

In the second meeting (October 18th, 2023), the results of the individual online voting, along with discussion reports, were used to define the live voting round. During this round, both the SB and the extended experts panel took part to facilitate sharing and discussions. All the scenarios were discussed, and each participant voted on the appropriateness of the interventions described using the same 9‐point numeric rating scale. In case of significant disagreement, the reasons behind the diverging opinions were discussed and efforts were made to appropriately modify the statements. If consensus was still not reached, the statement was rejected. If the average score remained above seven, an agreement was reached. If the score falls below seven, the discussion focuses on potential modifications to the statement. After the modification was agreed upon during the live session, the statement was voted on one last time.

## 3 RESULTS

A mean of 43 (±4, SD; range: 31–45) experts took part in the first round of voting. Participants were reduced to 38% of those originally selected (mean ± SD: 28 ± 2; range: 25–32) in the second round. All the physicians (*n* = 20) and caregivers representatives joined all the Delphi rounds. After the two‐round discussion, no statements were eliminated. The final 16 statements covered four major needs and issues associated with managing rare epilepsies, namely: epidemiology/nosology; diagnosis and prognosis; therapeutic management and multidisciplinary care. Ten statements reached a level of achievement (mean ± SD: 8.14 ± 0.42) sufficient to consider them approved already at the first voting round. Following some minor amendments, all the statements were approved (lower: 7.0; maximum: 8.9). The full list of the statements with the values obtained during the first and second voting rounds is reported in Table 1.

**TABLE 1:** List of Delphi statements.

Statement		First round	Second round
		Mean value ± SD	Mean value ± SD
Epidemiology/nosology	1. DEEs are diseases with variable etiologies in which epilepsy contributes to neurological and/or cognitive deficits	7.78 ± 2.0	7.9 ± 2.0
	2. The prevalence of DEEs, such as Dravet (1:45.000) or CDKL5 (1:50.000), is based on available databases and is reliable *Revised as*: The prevalence of DEEs, such as Dravet (1:45.000) or CDKL5 (1:50.000), is based on available databases but is not correctly estimated	5.4 ± 2.5	7.0 ± 1.7
	3. The mortality of DEEs (2%–5%) is overestimated *Revised as*: The mortality of DEEs (2%–5%) is not correctly estimated	5.23 ± 1.9	7.7 ± 0.8
Diagnosis and prognosis	4. Etiology influences the therapeutic management and outcome of epilepsy	8.18 ± 0.9	8.3 ± 1.0
	5. The therapeutic management may aggravate the outcome	7.96 ± 1.2	8.3 ± 0.8
	6. During adolescence or adult age improvement of epilepsy is common *Revised as*: During adolescence or adult age improvement of epilepsy is possible	6.2 ± 1.8	8.0 ± 0.8
	7. Status Epilepticus (including non‐convulsive SE) or incoming crisis are frequent	7.36 ± 1.1	7.9 ± 0.9
	8. Controlling tonic–clonic seizures and drop seizures is the first goal to impact the Quality of Life (QoL) *Revised as*: Between the possible types of seizures, controlling tonic–clonic and drop seizures is the first goal to impact the Quality of Life (QoL)	7.18 ± 1.3	8.4 ± 0.7
Therapeutic management	9. Pharmacological treatment supposes the early use of drugs with a specific indication (e.g., stiripentol, felbamate, fenfluramine, cannabidiol)	8.2 ± 0.9	8.6 ± 0.6
	10. Non‐pharmacological treatments should be considered after the failure of, at least, 2 anti‐seizure medications *Revised as*: Non‐pharmacological treatments could be considered already after the failure of 2 anti‐seizure medications	6.98 ± 1.8	7.2 ± 1.9
	11. The introduction of a second or/and third anti‐seizure medication should consider the effects on comorbidities	8.24 ± 1.1	8.7 ± 0.6
Multidisciplinary care	12. The behavioral and cognitive abnormalities represent the most relevant problem in the adolescent/adult age	8.02 ± 0.9	7.8 ± 1.3
	13. Standardized scales, coherent with the age and native language of the patient, should be used for rating the cognitive‐behavioral comorbidities	8.2 ± 1.0	8.7 ± 0.7
	14. Continuity of care is a process engaging the hospital and territorial services together, taking into consideration the cognitive and behavioral comorbidities of every single patient to build a coherent “plan of life”	8.69 ± 0.6	8.9 ± 0.5
	15. Transition is not only a “handover” between clinicians of the pediatric and adult age	8.69 ± 0.6	8.9 ± 0.4
	16. A multidisciplinary approach is the sole possibility for properly taking charge of patients	8.82 ± 0.5	8.9 ± 0.3

*Note*: Statements are listed in numerical order and divided into “fields.” On the right, the mean ± SD scores obtained at each voting round are reported.

### 3.1 Epidemiology/nosology reveals the impact of epilepsy on the overall disease phenotype and unreliable prevalence data in the literature

**FIGURE 1:**
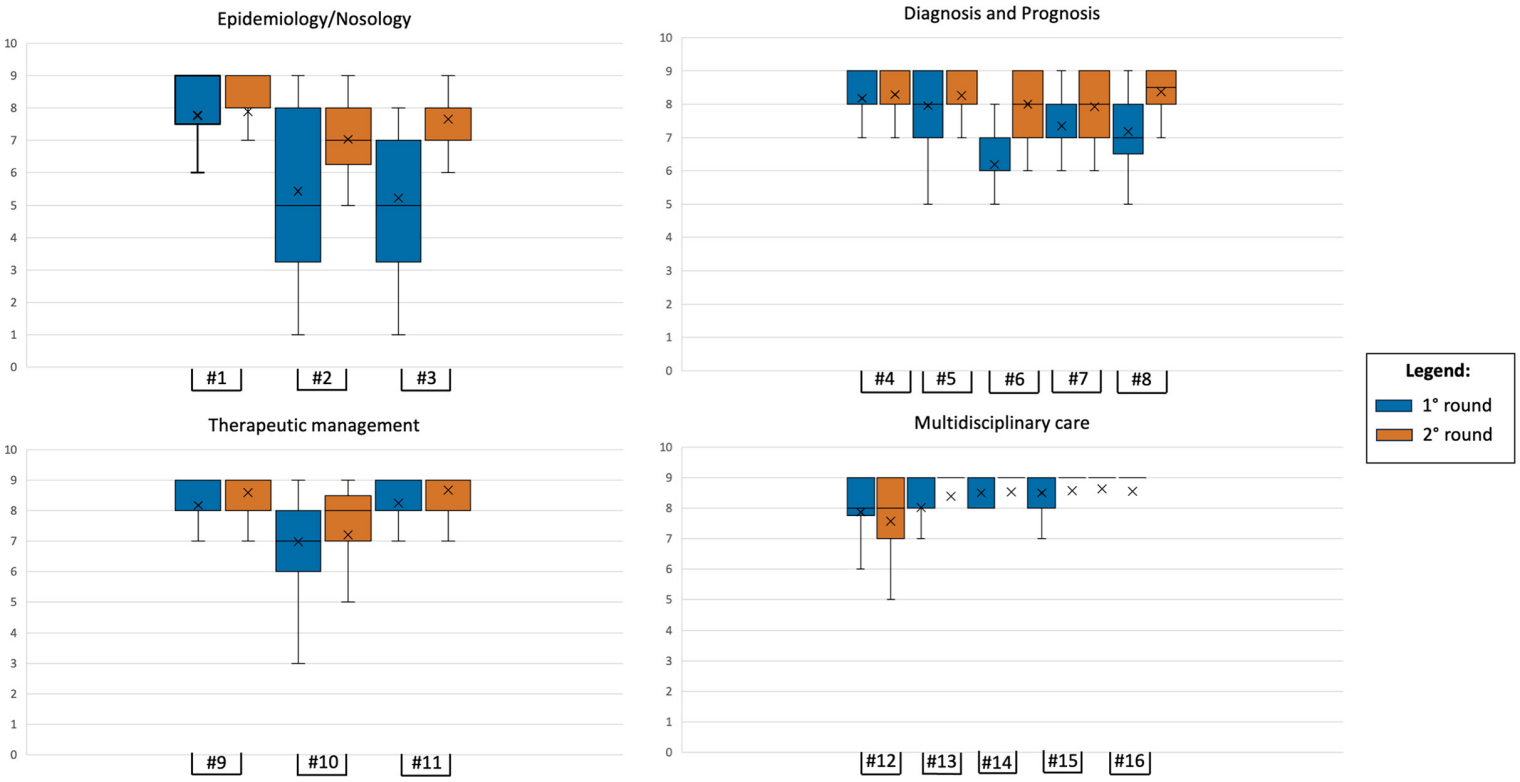
Graphical representation of the mean ± SD scores for each statement. Box‐and‐whisker plots help visualize the summary of the consensus for each statement. Statements are in numerical order (#number) and divided based on the “area” they belong to. Two different colors are used to represent the first and second voting round.

For statement #1, dealing with the etiological heterogeneity of these disorders and the impact of epilepsy on the overall outcome, consensus was reached at the first voting round. The consensus of the experts' panel was confirmed at the second voting round. Conversely, disagreement on the formulated statements #2 and #3 required their reformulation in Round 2. Specifically, both statements dealt with the estimates for disease prevalence and mortality available in current databases/in the literature (Figure 1).

### 3.2 Etiology is the main determinant for prognosis, but some phenotypic manifestations are invariably impactful between DEEs

Panelists agreed on identifying etiology as the driver for therapeutic choices and outcome in DEEs (statement #4); as a matter of fact, a misdiagnosis may lead to therapies with a negative impact on the disease course (statement #5). Prolonged seizures and SE (both convulsing and non), as well as generalized tonic–clonic seizures, are the main concerns for the caregiver, influencing the perceived QoL. Conversely, when children grow up epilepsy may tend to spontaneously improve, and the burden of non‐seizure outcomes becomes relevant (statements #6, #7, #8) (Figure 1).

### 3.3 Specific pharmacological and non‐pharmacological interventions should be implemented early

Statement #9 stressed the importance of the early use of anti‐seizure medications (ASMs) with specific indications for DEEs, and the panelists agreed on that. In the last few years, diverse ASMs (e.g., fenfluramine, cannabidiol, ganaxolone) have received approval from regulatory agencies for the labeled use in some DEEs. Although the mechanisms of action are not etiology‐driven, these drugs have shown to be impactful on the neurodevelopmental trajectories^22^ and the non‐seizure outcomes^23^, ^24^ as opposed to “classic” ASMs (e.g., topiramate and valproate) which are known to cause lowered cognitive/neuropsychological performances.^25^ Consequently, clinicians agreed that a fundamental “shift” in the therapeutic algorithm for rare and complex epilepsies is needed. Moreover, another important point needs to be considered: non‐pharmacological interventions (e.g., vagal nerve stimulation) should be implemented early in the therapeutic management of patients, just after the failure of two appropriately chosen and dosed ASMs. This was highlighted by the slight disagreement on statement #10, which then reached an agreement after revision. Finally, statement #11 remarked on the importance of comorbidities in the overall therapeutic management of patients (Figure 1).

### 3.4 Multidisciplinarity allows a holistic approach to care of patients

A high agreement was reached among panelists for statements dealing with the need for a multidisciplinary approach to DEEs. The mean score of statements #12, #13, #14, #15, and #16 was 8.48 ± 0.35 already at the first voting round. The mean score increased to 8.64 ± 0.5 in the 2nd round, confirming general agreement on the topic (see Figure 1; Table 1).

## 4 DISCUSSION

Developmental Encephalopathies and Developmental and Epileptic Encephalopathies are known to be two distinct entities.^2^, ^3^ Also, their biological basis is highly heterogeneous, with some cases caused by pathogenic variants in genes involved in early brain development, others caused by nongenetic etiologies (i.e., structural, metabolic, or infectious), and, finally, “undefined” (also called “idiopathic”) cases, which are worthy further investigations. However, the impact of epilepsy and EEG abnormalities on the course of the disease is well established, even if hardly measurable.^26^

The low mean scores ± high SD obtained for statements evaluating “Epidemiology/Nosology,” underline the need for unique disease registries and epidemiological studies in the care of patients with complex and rare epilepsies. Particularly, registries will not only help physicians to provide families with clear estimates of the disease affecting their children/assisted persons, but they will also help researchers in studying the pathophysiology and potential therapeutic targets of the diseases. For example, a registry for Dravet Syndrome (Residras)^27^ has been implemented through the help of patient associations and is currently recruiting patients on the national territory. DS patients reveal a spectrum of clinical manifestations; both seizures, cognition and motor impairment have a variable severity from patient to patient and overtime with only a slight genotypic correlation. *SCN1A* pathogenic variants, identified in more than 80% of patients, do not explain such heterogeneity that may be due to other genetic “susceptibilities”.^28^, ^29^

Dissecting these boundaries becomes fundamental to developing targeted therapies and will add the values given by registries. Similarly, International and National registries for CDKL5 deficiency disorder (CDD), AS, TSC and LGS are also actively recruiting patients, and Natural History studies, specifically informing about developmental trajectories, are being carried out.^30^ These are common venues in most DEEs; correct epidemiological data will lead to better resource allocation and tailored public health strategies. Moreover, these registries may help guide healthcare professionals in diagnosis and management, and inform policymakers about the needs of this patient population.

The “Diagnosis and Prognosis” area, covered by our national consensus, reinforces the etiological complexity underlying rare epilepsies and the need for proper classification to guide therapeutic choices and inform patients/caregivers on the supposed progression of the disease. However, there was generally more consensus on the given statements with mean scores already passing seven at the first voting round. More discussion derived from the supposed improvement of epilepsy during adulthood and the burden of seizures. DEEs are invariably different yet some commonalities can be found, specifically, caregivers are in line in stressing the relevance of epilepsy and the high impact of prolonged, generalized tonic–clonic and drop seizures over the QoL. In addition, recognizing the negative impact of certain therapies on outcomes also stresses the need for pointful and careful medication management and monitoring.

The therapeutic management of DEEs is mostly based upon algorithms^31^, ^32^, ^33^, ^34^; although, the choice of *add‐on* medications strictly depends on the physician's experience and way of working. Panelists agreed that drugs with a specific indication should be considered early in the diagnostic algorithm. Similarly, non‐pharmacological treatments should not be delayed for multiple ASM testing. Indeed, the common trend is to add specific therapies or consider non‐pharmacological strategies when all the first‐line pharmacological options have failed. This should change in the next few years thanks to the increasing relevance of ASMs with robust supporting studies and highly reliable results of efficacy in some DEEs. These drugs, together with non‐pharmacological treatments, are commonly not directly targeting the biological mechanisms of the disease, but have the potential, if moved from second‐ to first lines, to favorably modify the outcome of these rare diseases.^35^, ^36^, ^37^ Keeping in mind “comorbidities,” or better “non‐seizure outcomes,” in treatment plans points towards a patient‐centric approach in Italy. This could encourage the adoption of newer, more holistic, medications and the integration of alternative therapies into the standard of care practices.

The need for a multidisciplinary approach to DEEs is well recognized in the literature,^38^ and the results of our consensus seem to convene that this approach has been already implemented in highly specialized Italian centers as highlighted by the high scores obtained in this area of the study (Figure 1). Multidisciplinarity implies changes in how teams are organized and coordinated between different specialties. A therapist, specializing in epilepsy, should always be part of the team to address not only the medical but also the cognitive, behavioral, and social aspects of epilepsy, for which standardized scales are pivotal. In addition, attention should be paid not only to allocating resources to ameliorate *transition*, which should be considered not only a “handover,” but also a complex process involving either the hospital or community services, to the end of building “life projects.”

This consensus reflects a progressive and comprehensive approach to epilepsy care in Italy, highlighting the need for personalized, holistic, and multidisciplinary care. These consensus statements may influence healthcare policies in Italy, advocating for more funding for epilepsy research, better training for healthcare professionals, and the development of specialized centers for epilepsy care. This could also lead to the formulation of new guidelines and protocols for the management of epilepsy, based on the latest scientific evidence and expert opinion. The focus on personalized care, the recognition of the multifaceted nature of epilepsy, and the emphasis on multidisciplinary approaches are likely to enhance patient satisfaction and outcomes. This could also empower patient communities, providing them with more information and support for managing their frail condition.

## 5 CONCLUSION

The integration of scientific evidence with expert judgment at the i‐RARE event highlights the importance of advancing medical practice, especially in epilepsy care, by combining empirical data with clinical experience for more informed decisions. This approach will enhance decision‐making, enable personalized medicine, bridge research and clinical practice, navigate complex scenarios, foster innovative treatments, and aid in policy development. It will ensure that epilepsy care is not only based on solid research but also tailored to individual needs and real‐world contexts, driving continuous improvement in patient care and policy frameworks. Moreover, engaging with patients and caregivers in research to understand their experiences will provide valuable insights for developing patient‐centered care models.

The consensus reached at the i‐RARE provides recommendations for clinicians, particularly in the diagnosis, treatment, and management of DEEs, helping to standardize care practices and improve the quality of care for patients. The emphasis on early and specific pharmacological treatments, alongside the recognition of non‐pharmacological therapies and the importance of considering comorbidities, is likely to shape treatment protocols. This could lead to more comprehensive treatment plans that include newer medications and therapies. Moreover, these consensus statements are poised to guide policymakers in resource allocation and funding, potentially leading to increased research in key areas and the establishment of specialized epilepsy centers. The focus on individualized treatment approaches promotes the adoption of personalized medicine, ensuring that policies and practices are more patient‐centered and consider psychological, cognitive, and social aspects of epilepsy.

The strong agreement on the necessity of a multidisciplinary approach suggests a shift towards collaborative and integrated care models, ensuring better coordination among various healthcare providers. This approach can significantly improve the management of epilepsy, leading to better patient outcomes and enhanced quality of life. Additionally, the insights from these statements can update educational and training programs for medical professionals, aligning them with the latest evidence and practices in epilepsy care.

There is also a significant need for the development of new therapeutic agents, specifically targeting different types of rare epilepsies. Research in neuropharmacology and innovative drug delivery systems could be pivotal in this regard. Alongside these, focusing on the comorbidities associated with rare epilepsies, such as cognitive and psychological disorders, will aid in the development of more holistic care models. A multifaceted approach to research and development is sought, and it is necessary to advance understanding and improve the management of rare and complex epilepsies.

## AUTHOR CONTRIBUTIONS

A.R: collection of data, drafting; A.C., F.B., A.V., I.B. M.E., F.D., S.L., S.M., A.L.N., G.D.G., I.B., K.S., T.P., F.M., A.G., C.dB., S.B., C.M., D.P., G.C., and N.S: supported in collecting the data, revision of the final draft; G.G and P.S: conception of the study, revision, and final approval of the manuscript. All authors agree to be accountable for all aspects of the work.

## FUNDING INFORMATION

No target funding is to be reported.

## CONFLICT OF INTEREST STATEMENT

AR has received honoraria and travel grants from PTC Therapeutics, Kolfarma Srl, UCB, Jazz Pharmaceuticals and Proveca Ltd. AC received speaker's honoraria from UCB, Angelini, and Jazz Pharmaceuticals, and has served as a scientific consultant for advisory boards for Takeda, UCB, and Jazz Pharma. FB received honoraria from UCB and Angelini Pharma, and has served for scientific advisory boards for Takeda, Angelini Pharma and UCB. SL has received speaker's or consultancy fees from Angelini Pharma, Eisai, GW Pharmaceuticals, Medscape, and UCB Pharma and has served on advisory boards for Angelini Pharma, Arvelle Therapeutics, BIAL, Eisai, GW Pharmaceuticals and UCB Pharma outside the submitted work. SL received research grant support from the Ministry of Health and the Ministry of University and Research outside the submitted work. SM received research grant support from the Ministry of health (MOH); has received personal compensation as scientific advisory board member for UCB, Jazz pharmaceuticals, and EISAI. IB received unconditional grants/reimbursements from Dravet Italia Onlus (of which she is president), Jazz Pharmaceuticals, UCB, Takeda and Biocodex. NS has served on scientific advisory boards for GW Pharma, BioMarin, Arvelle, Marinus, and Takeda; has received speaker honoraria from Eisai, BioMarin, Livanova, Sanofi, Jazz Pharmaceuticals, UCB, Takeda; has served as an investigator for Zogenix, Marinus, Biomarin, UCB, Roche. PS received speaker's honoraria from UCB, Angelini, and Jazz Pharmaceuticals, and has served as a scientific consultant for advisory boards for Biomarin, Takeda, Proveca, UCB, and Jazz Pharmaceuticals. None of the other authors has any conflict of interest to disclose. We confirm that we have read the Journal's position on issues involved in ethical publication and affirm that this report is consistent with those guidelines.

## ETHICS APPROVAL STATEMENT

All methods were performed in accordance with the ethical standards as laid down in the Declaration of Helsinki and its later amendments or comparable ethical standards.^1^

## Data Availability

The data that support the findings of this study are available in the article. If additional data were required, they might be requested to the corresponding author.

## APPENDIX 1

### 1.1

iRARE Study Group: Bartolini Emanuele, (IRCCS Stella Maris Foundation, Pisa, Italy); Bonanni Paolo, (IRCCS Eugenio Medea Scientific Institute, Epilepsy Unit, Conegliano); Boni Antonella, (IRCCS Istituto delle Scienze Neurologiche di Bologna, UOC Neuropsichiatria Dell'età Pediatrica, Bologna, Italy); Briatore Eleonora, (Department of Pediatric Neurology, Santa Croce e Carle Hospital, Cuneo, Italy); Canafoglia Laura, (Neurophysiopathology, Fondazione IRCCS Istituto Neurologico Carlo Besta, Milan); Del Negro Ilaria, (Clinical Neurology Unit, Udine University Hospital, Piazzale S. Maria della Misericordia, 33100 Udine, Italy. Department of Medical Area (DAME), University of Udine, 33100 Udine, Italy); Fortunato Francesco, (Department of Medical and Surgical Sciences, Institute of Neurology, University Magna Graecia, Catanzaro, Italy); Galli Rosita, (Azienda Usl Toscana Sud Est UOC Neurologia Arezzo –Valdarno); Giordano Lucio, (Child Neurology and Psychiatry Unit, Spedali Civili, Brescia); Giugno Alessia, (Department of Medical and Surgical Sciences, Institute of Neurology, Magna Graecia University of Catanzaro, Catanzaro, Italy); Giuliano Loretta, (Department “G.F. Ingrassia”, Section of Neurosciences, University of Catania, Catania, Italy); Lavra Loredana, (Centro per i Disordini del Movimento, Dipartimento di Scienze Cardiovascolari e Neurologiche, Sezione Neurologia, Policlinico Universitario, Università di Cagliari, Cagliari); Luchetti Anna, (“Sapienza” University of Rome, Sant'Andrea Hospital, Via di Grottarossa, 1035‐1039, 00189 Rome, Italy); Maretti Michela, (Department of Diagnostic Medicine, Clinical and Public Health, Università degli Studi di Modena e Reggio Emilia); Marino Simona Domenica, (University Hospital “Policlinico‐San Marco”, Catania, Italy); Mastrangelo Mario, (Department of Women/Child Health and Urological Science, Sapienza University of Rome, Rome, Italy; Unit of Child Neurology and Psychiatry, Azienda Ospedaliero Universitaria Policlinico Umberto, Rome, Italy); Messana Tullio, (Infantile Neuropsychiatry, IRCCS – Institute of Neurological Sciences, Bologna, Italy); Parente Eliana, (Pediatric Neurology Unit and Epilepsy Center, Fatebenefratelli Hospital, ASST Fatebenefratelli Sacco, Milano, Italy); Pellino Giuditta, (Department of Medical Sciences‐Pediatric Section, University of Ferrara, Ferrara, Italy); Peruzzi Cinzia, (Child and Adolescent Neuropsychiatry Unit, Fondazione IRCCS San Gerardo dei Tintori, Monza, Italy); Piccioli Marta, (UOC of Neurology, PO San Filippo Neri, ASL Roma 1, Rome, Italy); Pietrafusa Nicola, (Clinical and Experimental Neurology, Full Member of European Reference Network EpiCARE, Bambino Gesù Children's Hospital, IRCCS, Rome, Italy); Pironi Virginia, (Department of Neuroscience, Bambino Gesù Children's Hospital, IRCCS, Full Member of European Reference Network EpiCARE, 00165 Rome, Italy. Center for Rare Diseases and Birth Defects, Department of Woman and Child Health, Institute of Pediatrics, Policlinico Universitario Gemelli Foundation, Catholic University of Rome, 00168 Rome, Italy); Prezioso Giovanni, (Department of Paediatrics, University of Chieti, Chieti, Italy); Prisco Giulia, (Alleanza Epilessie Rare e Complesse); Pulitano Patrizia, (Department of Human Neurosciences, Policlinico Umberto I, Sapienza University of Rome, Rome, Italy); Ragona Francesca, (Department of Pediatric Neuroscience, Fondazione IRCCS Istituto Neurologico C. Besta, Milan, Italy); Rizzi Romana, (Neuromotor & Rehabilitation Department, Neurology Unit, Azienda USL‐IRCCS of Reggio Emilia, Reggio Emilia, Italy); Rosati Eleonora, (Department Neurology 2, Careggi University Hospital, Florence, Italy); Ruta Maria Rosita, (UOC di NPIA ASP 6, Palermo); Sarajlija Jasenka, (Pediatric Neurology Unit, Infermi Hospital Rimini, Rimini, Italy); Spagnoli Carlotta, (Child Neurology Unit, Department of Pediatrics, Azienda USL‐IRCCS, Reggio Emilia, Italy); Spalice Alberto, (Department of Paediatrics, Child Neurology and Psychiatry, Sapienza University of Rome, Rome, Italy); Terrone Gaetano, (Department of Translational Medical Sciences, Università degli Studi di Napoli “Federico II”, 80125 Naples, Italy); Trabacca Antonio, (Scientific Institute IRCCS “E. Medea”, Unit for Severe Disabilities in Developmental Age and Young Adults (Developmental Neurology and Neurorehabilitation), Brindisi, Italy); Valerio Massimo, (Department of Medical Science, Università degli Studi di Torino); Zucca Claudio, (Department of Pediatric Neurology, Santa Croce e Carle Hospital, Cuneo, Italy).
